# 519. Risk Factor Analysis for Hospital Admission Following *Severe Acute Respiratory Syndrome Coronavirus-2 (SARS-CoV-2*) Monoclonal Antibody Treatment

**DOI:** 10.1093/ofid/ofab466.718

**Published:** 2021-12-04

**Authors:** Lana Abusalem, Cole Wood, Juan Carlos Rico Crescencio, Ryan K Dare

**Affiliations:** University of Arkansas for Medical Sciences, Little Rock, Arkansas

## Abstract

**Background:**

The FDA has issued emergency use authorization (EUA) for neutralizing monoclonal antibodies (mAb) for the treatment of mild-moderate coronavirus disease 2019 (COVID-19) in patients who are at high risk of disease progression. The EUA allows for COVID-19 mAb infusion to occur up to 10 days from symptom onset and due to logistics, mAb treatment typically occurs later in this 10 day window. Efficacy of early versus late mAb treatment is unknown

**Methods:**

In this single center, retrospective case-control study, we performed a risk factor analysis of patients with mild COVID-19 infection treated with mAb on the composite outcome of subsequent evaluation in the Emergency Department (ED) or inpatient admission December 2020 through May 2021. Multivariate analysis of variables found to be significant in univariate analysis was performed using STATA 15 statistical software

**Results:**

Two-hundred eighty-eight patients who received mAb treatment were included in analysis. The mean age was 58.6 years and 59.7% were female, 64.9% white, and 27.1% African American. Following mAb infusion, 31 (10.8%) had disease progression resulting in an ED encounter or inpatient admission. Patients who received early (days 1-5 of symptoms) mAb infusion were less likely to have progressive disease than patients with late (days 6-12 of symptoms) infusion; (6.1% vs 13.2%; P= 0.048). Zero of 21 patients who received mAb infusion on day 1-3 of symptoms had disease progression. Patients with CHF (7.4% vs 19.4%; P=0.038), cirrhosis (9.3% vs 25.8%; p=0.012), CKD (12.5% vs 35.5%; p=0.001) and hypertension (70.8% vs 90.3%; p=0.021) were more likely to have disease progression. There were no differences in sex, race, BMI, or symptoms between groups. Multivariate analysis revealed cirrhosis (OR 3.0; 95% CI 1.1-7.9) and CKD (OR 2.6; 95% CI 1.0-6.4) increased risk of disease progression while early mAb infusion was protective (OR 0.38; 95% CI 0.14-1.0)

**Conclusion:**

Infusion of mAb for the treatment of mild to moderate Covid-19 within 5 days of symptom onset reduces rate of disease progression compared to delayed (day 6-12 of symptoms) infusion. This finding was significant when controlling for comorbidities. Efforts should be made to infuse high risk patients with COVID-19 mAb therapy within 5 days of symptom onset

**Disclosures:**

**All Authors**: No reported disclosures

